# Examining First Night Effect on Sleep Parameters with hd-EEG in Healthy Individuals

**DOI:** 10.3390/brainsci12020233

**Published:** 2022-02-08

**Authors:** Ahmad Mayeli, Sabine A. Janssen, Kamakashi Sharma, Fabio Ferrarelli

**Affiliations:** Department of Psychiatry, University of Pittsburgh, Pittsburgh, PA 15213, USA; mayelia@upmc.edu (A.M.); janssensa@upmc.edu (S.A.J.); kas521@pitt.edu (K.S.)

**Keywords:** first night effect, high-density EEG, sleep, sleep architecture, sleep power spectra

## Abstract

Difficulty sleeping in a novel environment is a common phenomenon that is often described as the first night effect (FNE). Previous works have found FNE on sleep architecture and sleep power spectra parameters, especially during non-rapid eye movement (NREM) sleep. However, the impact of FNE on sleep parameters, including local differences in electroencephalographic (EEG) activity across nights, has not been systematically assessed. Here, we performed high-density EEG sleep recordings on 27 healthy individuals on two nights and examined differences in sleep architecture, NREM (stages 2 and 3) EEG power spectra, and NREM power topography across nights. We found higher wakefulness after sleep onset (WASO), reduced sleep efficiency, and less deep NREM sleep (stage 3), along with increased high-frequency NREM EEG power during the first night of sleep, corresponding to small to medium effect sizes (Cohen’s d ≤ 0.5). Furthermore, study individuals showed significantly lower slow-wave activity in right frontal/prefrontal regions as well as higher sigma and beta activities in medial and left frontal/prefrontal areas, yielding medium to large effect sizes (Cohen’s d ≥ 0.5). Altogether, these findings suggest the FNE is characterized by less efficient, more fragmented, shallower sleep that tends to affect especially certain brain regions. The magnitude and specificity of these effects should be considered when designing sleep studies aiming to compare across night effects.

## 1. Introduction

Adaptation nights are commonly implemented in sleep studies to counter differences in baseline sleep data caused by environmental factors. One of these environmental factors is the first night effect (FNE). The FNE is an alteration in sleep characteristics observed during the first night of sleep studies, and it has been initially assessed by examining changes in sleep architecture parameters [1,2,3]. The main findings of these studies included lower sleep efficiency, an increase in wakefulness after sleep onset, and an overall decrease in the duration of sleep [1,4,5,6]. Furthermore, the FNE has been observed to induce significant changes in non-rapid eye movement (NREM) sleep parameters, such as delayed NREM sleep latencies during the first night of sleep [7].

In addition to sleep architecture, some studies have examined FNE on sleep EEG activity, especially during the deepest stages of NREM sleep (stage 3; N3), also referred to as slow-wave sleep (SWS) [8]. SWS is characterized by large, high amplitude slow EEG activity in NREM sleep, which is also described as slow wave sctivity (SWA, 1–4 Hz) [9]. During SWS, our brain becomes less sensitive to any external stimulus; as a result, it is considered the deepest stage of sleep as it is the most difficult stage from which to awaken. In a recent sleep study, Tamaki et al. reported reduced SWA during the first night of sleep in 10 healthy individuals [10]. Higher spectral power within alpha and beta bands was observed in SWS during the first night relative to the second night in healthy individuals [11]. Furthermore, another sleep study reported that theta and sigma NREM sleep EEG activity tended to be lower on the first night of sleep compared to the following nights [12].

The vast majority of the existing FNE studies have not systematically examined differences in both sleep architecture and NREM sleep EEG power spectra in study participants. Furthermore, all the previous FNE studies have analyzed sleep data acquired by a low-density EEG set-up, ranging from 2 to 19 electrodes [11,13,14,15]. In contrast, the more recent availability of high-density EEG (hd-EEG, N ≥ 64 channels) has allowed collecting EEG data with enhanced spatial resolution. Thus, hd-EEG is a useful tool for spatial analyses of scalp EEG activity and the topographical representations of EEG activity recorded during sleep [16]. Moreover, hd-EEG can be utilized to provide information about the topographical characteristics of differences in sleep parameters across a certain condition that is being examined (i.e., FNE in healthy individuals). Given that the FNE can influence sleep in various ways, including showing local effects on sleep EEG activity, it is important to assess its overall impact on sleep characteristics, from sleep architecture parameters to sleep EEG power spectra and power topography.

To begin addressing these issues and to provide for the first time a characterization of the FNE on sleep characteristics, from architecture to EEG topographic power, in the present study, we performed hd-EEG recordings in healthy control individuals for two nights, and examined differences in sleep architecture, NREM EEG power spectra, and NREM power topography across two nights of sleep in the sleep laboratory.

## 2. Materials and Methods

### 2.1. Participants

A total of 27 healthy individuals (*n* = 27; 15 female), who had no current DSM psychiatric diagnosis as well as no personal history of mental illness or a family history of a psychotic disorder or bipolar illness, were recruited for the present study. These individuals were all between the ages of 14 to 34 (mean: 21.26; standard deviation: 4.62; 6 adolescents), had no medical or neurological illness, and were screened for significant head injuries that may have affected the central nervous system. Furthermore, eligible participants were taking no psychotropic medication at the time of their enrollment and participation.

### 2.2. Recruitment and Eligibility

Study participants were recruited mainly through Pitt + Me, an online registry developed by the clinical and translational science institute at the University of Pittsburgh. Individuals use this platform to show interest in specific studies and request to be contacted directly by the research team. They were also recruited through online and physical advertisements in the local community. Participants were financially compensated for their involvement in the study.

Study subjects completed comprehensive screening assessments at the western psychiatric hospital (WPH), including a structured clinical interview for DSM-IV disorders (SCID-IV) and the childhood brain injury interview. They also completed the Wechsler Abbreviated Scale of Intelligence (WASI) to help rule out intellectual developmental disorders that could hinder their ability to understand what was being asked of them. Other exclusion criteria included current pregnancy, history of alcohol or substance use in the past 12 months, and disorders that may have prevented participants from truly understanding the tasks (e.g., intellectual disability and/or a neurological disorder). The present study was approved by the University of Pittsburgh institutional review board, and all participants provided written informed consent prior to completing study procedures.

### 2.3. Sleep hd-EEG Recordings

Participants were asked to complete two nights of sleep at the University of Pittsburgh medical center WPH sleep clinic. Upon consenting to the study, they were asked about their sleep habits, including what time they usually go to sleep and wake up. Our staff accommodated these times to ensure people were sleeping as close to as they regularly do as possible. Specifically, participants were told to arrive at the sleep laboratory at least 1.5 h prior to their usual bedtime so they had enough time to be fitted with the EEG cap. Participants were then given a tour of the control room where the sleep technicians stay overnight, and the bedroom where they slept (during their first night visit). The sleep clinic is a shielded room so that there is no signal interference. Participants were also asked to turn their phones off during the night so nothing could interfere with the EEG signal. The bedroom where they slept was also equipped with a call button to summon a sleep tech or researcher, and a private bathroom. Whole night sleep hd-EEGs were collected in each study participant for both Night 1 and Night 2, and two electrodes were applied to the chin to record electromyography (EMG). A 128-channel EEG system (Electrical Geodesics INC., EGI, Eugene, OR, USA) was utilized to perform these overnight sleep EEG recordings. EEG data was originally recorded using Cz as a common reference at a sampling rate of 250 Hz. A conductive gel was applied to each electrode in order to obtain impedance values below 100 KΩ for all channels. Since sleeping for the first time in a novel environment may alter the participants’ sleep, this first night of sleep was compared to a second sleep night. To score the data, we used a six-channel montage (F3, F4, C3, C4, O1, and O2) along with one EMG channel that were applied separately and synchronized with the EEG amplifier as well as two channels located on the upper right canthus and lower left canthus of the eye to monitor for eye movements. Overnight sleep recordings were scored using the American Academy of Sleep Medicine (AASM) criteria [17] by certified sleep technicians.

### 2.4. Sleep hd-EEG Data Processing

MATLAB (The MathWorks Inc., Natick, MA, USA) was used to analyze the sleep hd-EEG data. Sleep EEG signals were first band-pass filtered between 0.5 Hz and 40 Hz and then downsampled to 128 Hz. EEG signals were then re-referenced to the average of all channels and divided into 6-s epochs for EEG power spectra calculation. We used Welch’s modified periodogram method in 2-s Hamming windows (with 50% overlap) to transform the EEG time series data into the frequency domain in the 0.5–40 Hz frequency range. To remove channels and epochs with high-frequency noise and/or other persistent artifacts (i.e., low-frequency drift due to poor channel contact or sweating), semi-automatic artifact rejection procedures were effectuated. Specifically, artifact rejection thresholds for low (1–4 Hz) and high (20–30 Hz) frequency ranges were automatically calculated at the 99.8th and 99.5th percentile, respectively, for each channel. We chose the higher threshold to account for muscle artifacts because muscle activity is usually in that frequency band. Furthermore, spectral power in these frequency ranges was plotted and visually inspected across all 6-s NREM epochs for every channel. Any channels in which artifacts affected most of the EEG recording were removed. We calculated average power spectra between 0.5 and 40 Hz with a 0.16 Hz resolution. Bad epochs rejection occurred through a separate automated algorithm that identifies epochs using a 36-sec moving window that has either 3 times higher than low frequency power or 6 times higher than the high frequency power of the average sliding window. The number of channels (M ± SD) excluded for each night was as follows: Night 1: 4.89 ± 3.46 and Night 2: 9.11 ± 4.38. The difference between the number of bad channels across the nights was due to more peripheral channels affected by artifacts and did not include any channels that showed significant power differences between the two nights. Further, the percentages of good NREM epochs for each night were as follows: Night 1: 79% ± 2% and Night 2: 79% ± 3%. NREM absolute EEG power from good channels and good epochs was then averaged across six frequency bands of interest, delta (1–4.5 Hz), theta (4.5–8 Hz), alpha (8–12 Hz), sigma (12–15 Hz), beta (15–25 Hz), and gamma (25–40 Hz), and compared between nights in study participants to assess for FNE.

The power spectra were calculated by averaging the power among all channels for each frequency band. We then performed topographic power analysis to assess for power difference among all individual channels between the two nights.

### 2.5. Statistical Analyses

Student’s paired *t*-tests were conducted to compare sleep architecture and NREM sleep EEG power spectra variables across nights. Mass-univariate statistics for each channel independently were used to compare EEG power topographies between nights for each frequency band (i.e., Delta, Theta, Alpha, Sigma, Beta, and Gamma). Multiple comparisons were carried out using threshold-free cluster-enhancement (TFCE; weighing parameters E = 0.5 and H = 2), followed by non-parametric maximum permutation statistics [18]. In sum, each channel statistic is individually examined for support from its neighboring channels, in which the statistic is enhanced if neighboring channels show a similar pattern of differences. These enhanced statistics are then subjected to a single permutation test whereby the allocation of each dataset to each parameter setting is randomized 10,000 times. This creates a single empirical null distribution for each channel to find its individual p-value (corresponding to the 5% of randomized permutations, which show a larger maximum statistic) [18]. We also calculated the Cohen’s d to determine the effect sizes (effect size, ES) of the significant sleep parameter differences between Night 1 and Night 2. Cohen’s d values indicate how different a parameter of interest is between nights [19]. Effect sizes were computed on the average values of the sleep parameters that were significantly different between Night 1 and Night 2.

## 3. Results

All 27 healthy individuals completed both nights. We were also able to collect and compare sleep architecture, sleep power spectra, and sleep EEG topographic power differences across nights in each study participant.

### 3.1. Sleep Architecture

We found differences in sleep architecture parameters across nights (See Table 1). Specifically, study participants showed decreased sleep efficiency (*p* = 0.020, effect size = −0.5), reduced NREM stage N3 sleep, also called slow-wave sleep (*p* = 0.033, effect size = −0.4), and increased waking after sleep onset (WASO, *p* = 0.018, effect size = 0.5) during the first night compared to the second night of sleep. Altogether, these findings point to a deeper, more efficient, and less fragmented sleep on Night 2. It should be noted that these across-night changes were no longer significant after correction for multiple comparisons. Furthermore, we found no across night difference in any of the other sleep architecture parameters, including total sleep time, sleep latency, and time spent in REM sleep.

### 3.2. Sleep EEG Power Spectra

EEG power spectra comparison across nights revealed that high-frequency activities, including sigma (*p* = 0.0297, effect size = 0.4), beta (*p* = 0.013, effect size = 0.5), and gamma (*p* = 0.031, effect size = 0.4), were all enhanced during Night 1 when compared to Night 2 (See Table 2). Again, these across-night differences did not survive multiple comparisons correction. Furthermore, there was less delta power, also referred to as slow-wave activity (SWA, 0.5–4 Hz) during Night 1 (23.888 ± 14.406) when compared to Night 2 (25.506 ± 16.010), although this decrease in SWA was not significant. We also found no across-night differences in theta and alpha NREM EEG power spectral activity (Table 2).

**Figure 1:**
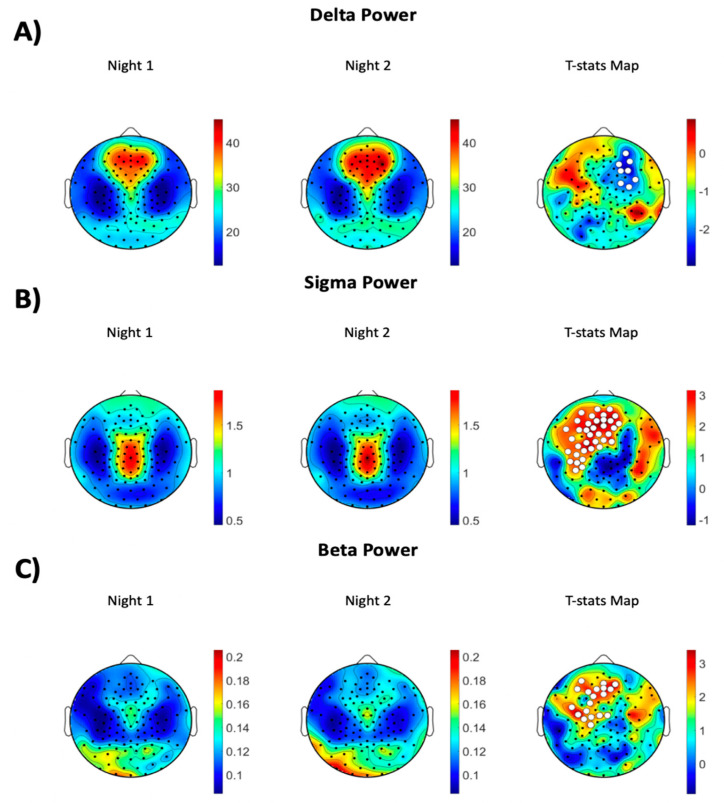
EEG power topographic maps during NREM sleep in (**A**) delta, (**B**) sigma, and (**C**) beta frequency bands. The left column shows topographic maps during Night 1, the middle panel, during Night 2, and the right panel across-night topographic t-stats statistical maps (Night 1 vs. Night 2). The white dots represent channels that had significantly different power across the two nights (paired statistical non-parametric mapping [SNPM]; corrected TFCE *p* < 0.05).

Topographic analyses revealed several important findings. First, we established that participants had lower SWA during the first night compared to the second night of sleep, which was localized in the right frontal/prefrontal brain region (cluster size = 8 channels, *p* = 0.046, effect size = −0.5, TFCE corrected, see Figure 1A). Furthermore, we found higher NREM sigma power (cluster size = 37 channels, *p* = 0.005, effect size = 0.8, TFCE corrected, see Figure 1B) and NREM beta activity (cluster size = 19 channels, *p* = 0.033, effect size = 0.7, TFCE corrected, see Figure 1C) during Night 1 compared to Night 2 in medial and left frontal/prefrontal regions. No significant differences were observed in the topographic map of any other frequency bands. Supplementary Figure S1 shows the EEG power topographic maps in nights 1 and 2 in theta, alpha, and gamma bands.

## 4. Discussion

By employing hd-EEG, the present study investigated the FNE on sleep architecture, NREM EEG power spectra, and NREM power topography in young, healthy individuals. Study participants showed higher wakefulness after sleep onset, reduced sleep efficiency, and less deep NREM sleep (i.e., SWS), along with increased high-frequency NREM EEG power during the first night of sleep. These between night differences in sleep architecture and NREM sleep EEG power spectra yielded small to medium effect sizes. Furthermore, these individuals showed significantly lower SWA, which characterizes the deepest NREM sleep, in the right prefrontal electrodes combined with higher sigma and beta activities in the medial and left prefrontal channels during the first compared to the second night of sleep. Across night topographic differences in NREM sleep EEG activity corresponded to medium to large effect sizes.

Overall, our sleep architecture findings showed that, during the first night spent in the laboratory, sleep was less efficient, more fragmented (i.e., higher WASO), and shallower (i.e., reduced N3 NREM sleep). Previous studies reported a lower sleep efficiency [11,20,21,22,23] and higher WASO [20,21,23] during the first night’s sleep, which is consistent with our results. Of note, these effects did not survive multiple comparisons; however, we still believe that these differences are relevant and that they should be considered when performing across conditions and/or across group comparisons in sleep architecture parameters. A possible FNE on these sleep parameters should also be considered when examining the effects of any intervention involving sleep manipulation and/or sleep enhancement in both healthy and clinical populations.

Another important finding of the present study was that, during the first night of sleep, the power spectra of the higher frequency bands (i.e., sigma, beta, and gamma) were enhanced when compared to the second sleep night. A higher level of activity in these fast frequency bands, which are more commonly observed during wakefulness, may reflect hyperarousability and/or increased alertness during sleep in order to ensure safety when sleeping in a new environment [21]. Indeed, during NREM sleep, both neuronal firing and gamma power tend to decrease, as does the ability to process sensory information [24,25]. Thus, a higher gamma activity in long-term meditators (LTM) could reflect a partially maintained capacity of parieto-occipital sensory and default network-associated areas to process information and maintain some level of awareness, even during a state when usually these cognitive functions are greatly impaired. An increase in fast frequency activity may also reflect a more disrupted, fragmented sleep. Consistent with this assumption, in a recent sleep study, we found that higher gamma activity was associated with WASO in both healthy and clinical groups [26]. While in this study, we found a higher average sigma power spectra as well as higher sigma power in right frontal/prefrontal areas, a previous sleep study conducted on eight healthy individuals found a lower sigma power in two specific channels [12]. Investigating a larger sample size and using hd-EEG with 128 channels with correction for multiple comparisons might account for this discrepancy.

By utilizing hd-EEG recordings during sleep, in this study, we were able to localize some of the effects observed in the power spectra analysis. For example, the increase in the sigma and beta frequencies that were found during the first night of sleep was especially present in the medial and left frontal/prefrontal regions. That is, study participants showed a decrease in these fast NREM sleep EEG activities from the first to the second night of sleep in these areas. Furthermore, we established an increase in slow NREM EEG activity, as reflected by higher SWA, that involved the right frontal/prefrontal region. SWA characterizes the deepest NREM sleep stage, tends to the maximal in frontal prefrontal areas, and has been extensively associated with the restorative function of sleep [27]. Thus, the combination of higher SWA and reduced fast activities in frontal/prefrontal areas indicates a deeper, more restorative sleep during the second night in the sleep laboratory. Notably, the increase in SWA was also present in the power spectra analysis but failed to reach significance. Previous sleep EEG studies have found substantial NREM sleep spectral power variability among healthy control participants, especially in the SWA range [28,29]. A large standard deviation in the SWA band was confirmed in the present study and may account for the less prominent and/or negative NREM power spectra effects relative to the NREM power topography results. While the sleep architecture and power spectra differences between two nights showed small to medium effect sizes, the topography differences exhibit medium to large effect sizes. Thus, these findings indicate that the FNE tends to be more prominent in specific brain regions. The present findings also suggest that the FNE effects may be localized rather than global and may affect especially certain brain regions, including frontal/prefrontal areas, that are more sensitive to sleep disruption [30].

Across night topographic changes in NREM SWA (i.e., lower SWA in Night 1 vs. Night 2) involved primarily one hemisphere. Interestingly, previous sleep studies in both humans [31] and animals [32,33] have reported lower SWA occurring in one hemisphere during the first night of sleep, which was then followed by a significant increase in SWA during the second night. Tamaki et al. (2016) reported a lower SWA in the left hemisphere in the default mode network (DMN) during the first night of sleep [31]. Here, we found that SWA is lower in the right hemisphere in Night 1 in a different brain region (i.e., frontal brain region). An intriguing possibility is that different regions in the two hemispheres may subserve the purpose of increased arousal and responsivity while sleeping for the first time in a novel environment. This FNE has been hypothesized to reflect a mechanism that is used to detect and protect from external stimuli when sleeping for the first time in an unfamiliar environment while still allowing for the other side of the brain to remain asleep. Our findings are consistent with this hypothesis, to the effect that lower SWA during Night 1 could be interpreted as reflective of an enhanced ability to detect and respond to outside input during sleep for the first time in a novel environment.

Future studies should investigate whether FNE is also present when study participants are allowed to sleep in their own home environment. Such future works will help establish with greater accuracy the effects of an unfamiliar environment (i.e., a sleep laboratory) on sleep parameters above and beyond the effects of undergoing novel and/or unusual procedures (i.e., performing a task before and after sleep, sleeping while wearing an EEG cap, etc.). Examining the EEG data before sleep (i.e., during wakefulness) may also help us understand the FNE better. Furthermore, having more than two nights of sleep in the sleep laboratory will help examine whether the adaptation to sleep in a novel environment may last more than one night. In this study, we focused on finding the FNE in NREM sleep; however, future studies should also investigate REM power topography differences across nights. Among the study participants, only six (out of 27) were minor, thus, making it unlikely to have any meaningful impact on our findings. Nonetheless, future work on larger groups of children/adolescents is needed to investigate possible differences in FNE in these groups. Finally, the FNE should be investigated in clinical populations to see if these individuals show the same effects observed in healthy subjects.

## 5. Conclusions

In this study, we performed high-density EEG (hd-EEG) sleep recordings on 27 healthy individuals on two nights and examined differences in sleep architecture, NREM EEG power spectra, and NREM power topography across nights. Altogether, these findings suggest the FNE is characterized by less efficient, more fragmented, shallower sleep that tends to affect especially certain brain regions. The magnitude and specificity of these effects should be considered when designing sleep studies that aim at comparing across night effects.

## Figures and Tables

**Table 1:** Sleep architecture parameters for nights 1 and 2 and statistical comparison across nights.

Variable	Night 1 (M ± SD)	Night 2 (M ± SD)	T-Stats	Uncorrected *p*-Value
**Total sleep** (min)	434.156 ± 78.558	448.426 ± 48.202	−1.111	0.277
**Sleep latency** (min)	21.433 ± 16.187	17.711 ± 14.342	1.696	0.102
**Sleep efficiency**	0.847 ± 0.131	0.906 ± 0.059	−2.475	0.020 *
**NREM 1** (min)	36.148 ± 16.576	31.130 ± 14.508	1.896	0.069
**NREM 2** (min)	221.493 ± 55.030	217.944 ± 37.704	0.430	0.671
**NREM 3** (min)	88.148 ± 35.559	100.370 ± 34.366	−2.252	0.033 *
**REM** (min)	88.367 ± 31.651	98.981 ± 23.056	−1.725	0.096
**WASO** (min)	57.326 ± 65.500	29.278 ± 28.475	2.512	0.018 *

Paired *t*-test was used to compare means of parameters in Night 1 vs. Night 2 among 27 healthy control individuals; * indicates significance; NREM = non rapid eye movement; REM = rapid eye movement; WASO = Wakefulness After Sleep Onset; M = mean; SD = standard deviation.

**Table 2:** NREM sleep EEG power spectra for nights 1 and 2 and statistical comparison across nights.

Variable	Night 1 (M ± SD)	Night 2 (M ± SD)	T-Stats	Uncorrected *p*-Value
**Delta**	23.888 ± 14.406	25.506 ± 16.010	−1.471	0.153
**Theta**	3.051 ± 1.605	2.981 ± 1.412	0.927	0.362
**Alpha**	1.326 ± 0.683	1.292 ± 0.617	0.678	0.504
**Sigma**	1.000 ± 0.531	0.967 ± 0.497	2.301	0.030 *
**Beta**	0.143 ± 0.047	0.137 ± 0.045	2.670	0.013 *
**Gamma**	0.0301 ± 0.009	0.028 ± 0.007	2.279	0.031 *

Paired *t*-test was used to compare means of parameters in Night 1 vs. Night 2 among 27 healthy control individuals; * indicates significance; M = mean; SD = standard deviation.3.3. Sleep EEG Topographic Power Differences.

## Data Availability

Data can be requested from the authors.

## Supplementary Materials

The following supporting information can be downloaded at: https://www.mdpi.com/article/10.3390/brainsci12020233/s1, Supplementary Figure S1. EEG power topographic maps during NREM sleep in (A) Theta, (B) Alpha, and (C) Gamma frequency bands.
