# Supplementary material for: Examining First Night Effect on Sleep Parameters with hd-EEG in Healthy Individuals
Source: Brain Sci. 2022 Feb 8;12(2):233. doi: 10.3390/brainsci12020233 (PMC8870064; doi:10.3390/brainsci12020233)
Supplement: Supplementary file 1 [file brainsci-12-00233-s001.zip › brainsci-1553703-supplementary.pdf]

Supplemental Materials for:

Examining First Night Effect on Sleep Parameters with hd-EEG in Healthy Individuals

Ahmad Mayeli, Sabine A. Janssen, Kamakashi Sharma, Fabio Ferrarelli*

Department of Psychiatry, University of Pittsburgh, PA 15213, USA

*** Corresponding Author:**

Fabio Ferrarelli, MD PhD
3501 Forbes Ave, Suite 456, Pittsburgh, PA 15213
Telephone Number: (412) 864-1668
Email: ferrarellif@upmc.edu

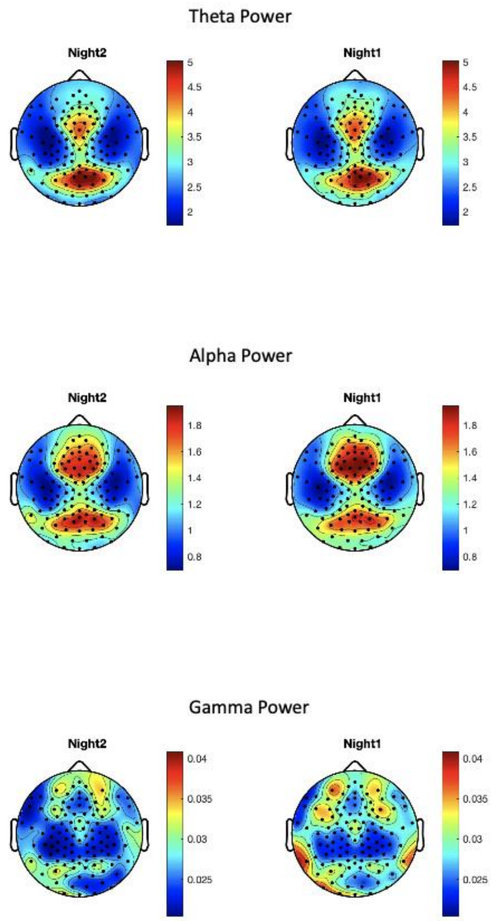

Supplementary Figure S1. Average EEG power topographic maps during NREM sleep in (A) Theta, (B) Alpha, and (C) Gamma frequency bands. The left column shows the topography map during night2, and the middle panel, night 1. It should be noted that no significant differences were found between the power spectra topography map in the aforementioned bands between 2 nights.
